# Functional Heterogeneity of CD4^+^ Tumor-Infiltrating Lymphocytes With a Resident Memory Phenotype in NSCLC

**DOI:** 10.3389/fimmu.2018.02654

**Published:** 2018-11-16

**Authors:** Anna E. Oja, Berber Piet, David van der Zwan, Hans Blaauwgeers, Mark Mensink, Sander de Kivit, Jannie Borst, Martijn A. Nolte, René A. W. van Lier, Regina Stark, Pleun Hombrink

**Affiliations:** ^1^Sanquin Research, Department of Hematopoiesis, and Landsteiner Laboratory, Amsterdam UMC, University of Amsterdam, Amsterdam, Netherlands; ^2^Department of Respiratory Medicine, Onze Lieve Vrouwe Gasthuis, Amsterdam, Netherlands; ^3^Department of Pathology, Onze Lieve Vrouwe Gasthuis, Amsterdam, Netherlands; ^4^Division of Tumor Biology and Immunology, The Netherlands Cancer Institute-Antoni van Leeuwenhoek, Amsterdam, Netherlands

**Keywords:** NSCLC, TRM, TILs, cytokines, exhaustion, differentiation, co-stimulation

## Abstract

Resident memory T cells (T_RM_) inhabit peripheral tissues and are critical for protection against localized infections. Recently, it has become evident that CD103^+^ T_RM_ are not only important in combating secondary infections, but also for the elimination of tumor cells. In several solid cancers, intratumoral CD103^+^CD8^+^ tumor infiltrating lymphocytes (TILs), with T_RM_ properties, are a positive prognostic marker. To better understand the role of T_RM_ in tumors, we performed a detailed characterization of CD8^+^ and CD4^+^ TIL phenotype and functional properties in non-small cell lung cancer (NSCLC). Frequencies of CD8^+^ and CD4^+^ T cell infiltrates in tumors were comparable, but we observed a sharp contrast in T_RM_ ratios compared to surrounding lung tissue. The majority of both CD4^+^ and CD8^+^ TILs expressed CD69 and a subset also expressed CD103, both hallmarks of T_RM_. While CD103^+^CD8^+^ T cells were enriched in tumors, CD103^+^CD4^+^ T cell frequencies were decreased compared to surrounding lung tissue. Furthermore, CD103^+^CD4^+^ and CD103^+^CD8^+^ TILs showed multiple characteristics of T_RM_, such as elevated expression of CXCR6 and CD49a, and decreased expression of T-bet and Eomes. In line with the immunomodulatory role of the tumor microenvironment, CD8^+^ and CD4^+^ TILs expressed high levels of inhibitory receptors 2B4, CTLA-4, and PD-1, with the highest levels found on CD103^+^ TILs. Strikingly, CD103^+^CD4^+^ TILs were the most potent producers of TNF-α and IFN-γ, while other TIL subsets lacked such cytokine production. Whereas, CD103^+^CD4^+^PD-1^low^ TILs produced the most effector cytokines, CD103^+^CD4^+^PD-1^++^ and CD69^+^CD4^+^PD-1^++^ TILs produced CXCL13. Furthermore, a large proportion of TILs expressed co-stimulatory receptors CD27 and CD28, unlike lung T_RM_, suggesting a less differentiated phenotype. Agonistic triggering of these receptors improved cytokine production of CD103^+^CD4^+^ and CD69^+^CD8^+^ TILs. Our findings thus provide a rationale to target CD103^+^CD4^+^ TILs and add co-stimulation to current therapies to improve the efficacy of immunotherapies and cancer vaccines.

## Introduction

T cells are important mediators of tumor immunity and T cell infiltration of most types of solid tumors is a favorable prognostic marker (1, 2). Immunotherapy boosting T cell functionality in tumors is rapidly gaining a foothold as standard treatment. Unfortunately, durable responses are only observed in a minority of patients (3), which is most likely related to the highly immunosuppressive microenvironment of most tumors. Moreover, there is growing awareness that not only the degree of tumor infiltration but also the composition of T cell infiltrates varies substantially even between patients with the same cancer. As in healthy tissues, it is unlikely that all subsets of T cells are equally adapted to the physiological properties of the tumor microenvironments. Understanding the composition of tumor infiltrating lymphocytes (TILs) and defining the populations that contribute most to anti-tumor responses is essential to boost efficacy of immunotherapy.

In the past few years it became clear that immunity in tissues requires adaptation to the physiological properties of those tissues. In both mice and humans a specific subset of memory T cells permanently resides in tissues. Effector and memory T cells first enter tissues as part of an antigen-specific response and subsequently take up residency and become resident memory T cells (T_RM_). Once established, T_RM_ are important for protecting barrier tissues against secondary infections (4). Due to their strategic location, T_RM_ can detect pathogens and kill infected cells at an early stage to control the spread of infection. As an effector mechanism T_RM_ produce effector molecules more rapidly than other memory T cells (5, 6). The rapid release of IFN-γ, TNF-α, and IL-2 primes the surrounding tissue and leads to the recruitment of auxiliary immune cells to the infected site (7, 8).

Different types of T_RM_ exist, residing in different tissues, but even within single organs strict spatial organization of T_RM_ subsets has been described (9, 10). As such, a subset of T_RM_ are specifically adapted for residence in epithelial tissues. These T_RM_ are traditionally characterized by the expression of CD69, which inhibits S1PR1 mediated egress from tissues (11), and CD103 (alpha subunit of αEβ7 integrin), which docks cells to epithelial E-cadherin (12, 13). Recently, a variety of novel markers have been revealed that characterize T_RM_. These include the chemokine receptor CXCR6, important for development of T_RM_ (14), and CD49a (α subunit of α1β1 integrin), necessary for retention and cytotoxic function of T_RM_ (15, 16). Another hallmark of T_RM_ is the expression of a broad range of inhibitory receptors. T_RM_ often reside in delicate tissues, thus their activation appears to be strictly regulated to prevent immunopathology (5, 6, 17).

In line with the epithelial origin of most solid tumors, varying numbers of infiltrating T cells with an intraepithelial CD103^+^ phenotype have been described. For several types of cancers, it is now appreciated that the presence of mainly CD103^+^CD8^+^ TILs is a positive prognostic marker (18–21). Among human NSCLC tumors with similar degrees of T cell infiltration, those with the greatest proportions of CD103^+^ cells have the best prognosis. These CD103^+^CD8^+^ TILs share gene expression programs and phenotypic properties of T_RM_, including the expression of CD69, CXCR6, and CD49a (21). T_RM_ characteristics of CD4^+^ TILs are less explored. Although, the necessity of CD4^+^ T cell help for the cytotoxic programming of CD8^+^ T cells is widely appreciated (22, 23), they have also been described to suppress tumor growth through the secretion of IFN-γ or direct killing of tumor cells (24, 25). While CD103^+^CD8^+^ TILs isolated from NSCLC demonstrated greater cytotoxic capacity toward tumor cells than their CD103^−^ counterparts (19), the functional characteristics of CD103^+^CD4^+^ TILs remain largely unexplored.

In this study we map the heterogeneity of CD4^+^ and CD8^+^ T cell infiltrates in human NSCLC and compare them with paired unaffected lung tissue. We investigated T_RM_ characteristics of TIL subsets and addressed the expression of various inhibitory receptors that can be targeted by checkpoint inhibition therapy. We demonstrated an increased number of CD103^+^CD8^+^ TILs in NSCLC compared to surrounding lung tissue. In contrast, numbers of CD103^+^CD4^+^ TILs were decreased. Although the highest expression of inhibitory receptors was found on CD103^+^ TILs this was paradoxical to the superior cytokine production especially of CD103^+^CD4^+^ TILs. While TILs producing effector cytokines had lower PD-1 expression than TILs not producing cytokines, TILs with high PD-1 expression produced CXCL13, indicative of functionally distinct subsets within TILs. Furthermore, we found TILs to have a less differentiated phenotype than lung T_RM_ and that additional co-stimulation enhances cytokine production of some TIL subsets. Understanding the properties of TILs with T_RM_ attributes may have important implications for future cancer treatments.

## Results

### Resident memory phenotypes in paired blood, lung and tumor samples of NSCLC patients

**Figure 1:**
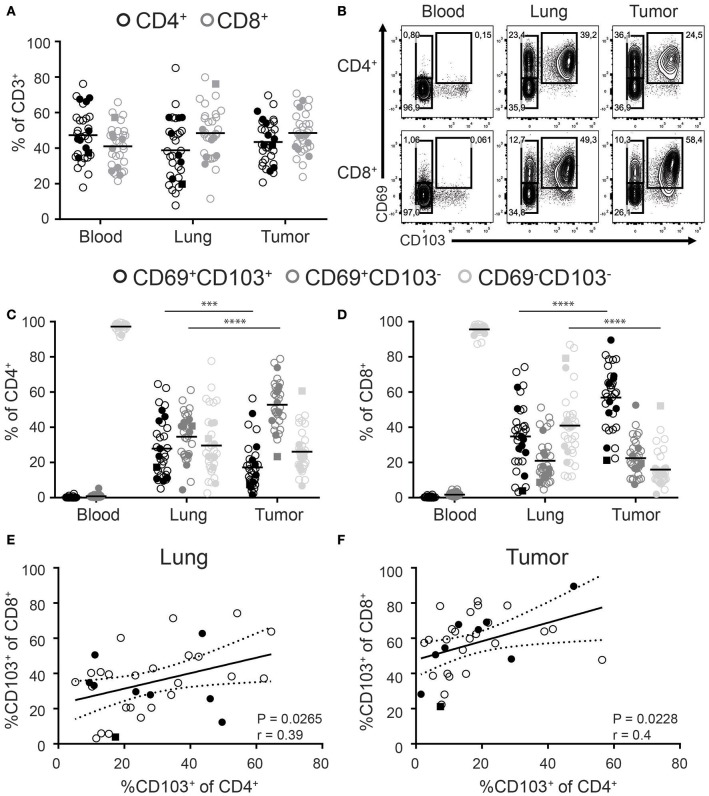
Distribution of CD103 and CD69 expression on CD4^+^ and CD8^+^ T cells of paired blood, lung, and tumor samples. **(A)** Frequencies of CD4^+^ (black circles) and CD8^+^ (gray circles) cells of total CD3^+^ T cells of paired blood, lung, and tumor tissue was analyzed by flow cytometry. **(B–D)** The expression of CD69 and CD103 was analyzed on paired blood, lung and, tumor CD4^+^ and CD8^+^ T cells. **(B)** Contour plots show representative examples of CD69 and CD103 expression on blood (left panels), lung (middle panels), and tumor (right panels) CD4^+^ (top panels) and CD8^+^ (bottom panels) T cells. **(C,D)** Frequencies of CD103^+^CD69^+^ (black circles), CD103^−^CD69^+^ (dark gray circles), and CD103^−^CD69^−^(light gray circles) cells of total blood, lung, and tumor CD4^+^ **(C)** and CD8^+^ **(D)** T cells were quantified. **(E,F)** Correlation between CD103^+^CD8^+^ and CD103^+^CD4^+^ lung **(E)** and tumor **(F)** T cells was determined. **(A–F)** *n* = 33. Open circles, solid circles, solid square indicate adeno-, squamous, and large cell carcinoma, respectively. **(A,C,D)** Quantifications are shown as dot plots with the horizontal line indicating the mean and each point represents a unique sample. **(E,F)** Correlation shown as X-Y graph where each point represents a unique sample. **(C,D)** ^***^p < 0.001, ^****^p < 0.0001; 2-way analysis of variance (ANOVA) with Tukey's multiple comparisons test. **(E,F)** r, Pearson's rank coefficient; *p* < 0.05.

While CD8^+^ T cells are in the spotlight of cancer immunotherapy, significant numbers of CD4^+^ T cells can also be found in solid tumors. We determined the frequencies of CD4^+^ and CD8^+^ T cells among the total CD3^+^ T cell pool in paired tumor, lung, and blood samples of 33 NSCLC patients. Included patients received a surgical resection of primary tumors as first line therapy without prior chemo- or radiotherapy. Blood was drawn from a central line at the start of surgery. We found comparable frequencies of CD4^+^ and CD8^+^ T cells in all three compartments (Figure 1A, general gating strategy in Supplementary Figure 1). Analysis of T_RM_ phenotypes was determined by the expression of CD69 and CD103. While CD69^+^ T cells were virtually absent in peripheral blood, they dominated in the lung and tumor (Figure 1B). In the blood, the frequency of CD103^+^ cells was low and as these cells lacked CD69 expression they cannot be defined as T_RM_ (data not shown). In contrast, both the lung and tumor compartments harbored high frequencies of CD69^+^CD103^+^ cells. As such, lung and tumor derived CD4^+^ and CD8^+^ T cells can be divided into three populations based on the expression of CD69 and CD103 (CD103^+^CD69^+^, CD103^−^CD69^+^, and CD103^−^CD69^−^; Figure 1B). For the rest of paper we refer to the CD103^+^CD69^+^ and CD103^−^CD69^+^ tumor and lung populations as CD103^+^ and CD69^+^ TILs and T_RM_, respectively, and tumor and lung CD103^−^CD69^−^ as CD69^−^ TILs and CD69^−^ T cells, respectively.

The percentage of CD103^+^CD8^+^ TILs was significantly increased compared to CD103^+^CD8^+^ lung T_RM_. The increased abundance of CD103^+^CD8^+^ TILs was accompanied by a decreased percentage of CD69^−^CD8^+^ TILs (Figure 1D). On the other hand, the decreased frequencies of CD103^+^CD4^+^ TILs was compensated by more CD69^+^CD4^+^ TILs (Figure 1C). Of note, while we included patients with different types of NSCLC (24 × Adeno-, 8 × Squamous, and 1 × Large cell carcinoma), no differences were observed in the frequency of the different subsets (Figure 1: Adeno—open circles, squamous solid circles, large cell carcinoma solid square). We further found a correlation between the frequencies of CD103^+^CD8^+^ and CD103^+^CD4^+^ in both the lung and tumor (Figures 1E,F).

### TIL populations are enriched for T cells with an early differentiated memory phenotype

**Figure 2:**
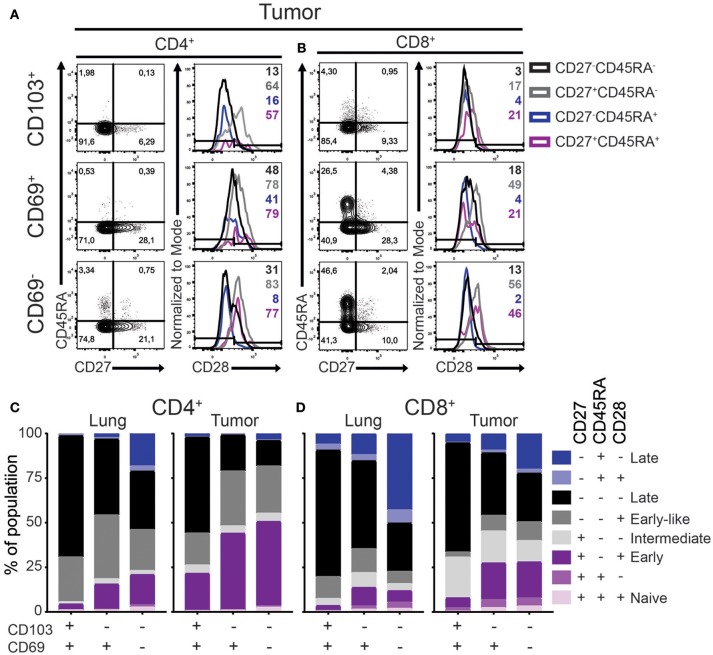
Differentiation status of lung T_RM_ and TILs. **(A–D)** The expression of CD45RA, CD27, and CD28 on CD4^+^ and CD8^+^ lung T_RM_ and TILs was determined. **(A,B)** The expression of CD27, CD45RA, and CD28 tumor CD103^+^ (top panels), CD69^+^ (middle panels), and CD69^−^ (bottom panels) CD4^+^ **(A)** and CD8^+^ **(B)** T cells shown by representative contour plots (CD45RA on y-axis, CD27 on x-axis) and histograms overlays (maximum set to 100%) show the expression of CD28 on the different subsets (black CD27^−^CD45RA^−^, gray CD27^+^CD45RA^−^, blue CD27^−^CD45RA^+^, purple CD27^+^CD45RA^+^). **(C,D)** The frequencies of CD27^+^CD45RA^+^CD28^+^ (light purple), CD27^+^CD45RA^+^CD28^−^ (medium purple), CD27^+^CD45RA^−^CD28^+^ (dark purple), CD27^+^CD45RA^−^CD28^−^ (light gray), CD27^−^CD45RA^−^CD28^+^ (medium gray), CD27^−^CD45RA^−^CD28^−^ (black), CD27^−^CD45RA^+^CD28^+^ (light blue), CD27^−^CD45RA^+^CD28^−^ (dark blue) of CD103^+^, CD69^+^, and CD69^−^ lung CD4^+^ (**C**; left bar graph), tumor CD4^+^ (**C**; right bar graph), lung CD8^+^ (**D**; left bar graph), and tumor CD8^+^ (**D**; right bar graph). **(C,D)** The quantifications are shown as bar graphs with the mean. *n* = 15.

A critical step in T_RM_ development is their recruitment into tissue where they undergo a process of maturation characterized by a loss of the co-stimulatory CD27 and CD28 receptors. We defined the differentiation stage of the different lung and tumor T cell subsets by analyzing the surface expression of CD45RA, CD28, CD27, and CCR7. While naïve T cells express all four markers, expression is lost stepwise by differentiating antigen-primed cells. Early, early-like, intermediate, late effector-type (CD45RA^−^) and late effector-type (CD45RA^+^) differentiated cells are described as, CCR7^−^CD27^+^CD45RA^−^CD28^+^, CCR7^−^CD27^−^CD45RA^−^ CD28^+^,CCR7^−^CD27^+^CD45RA^−^CD28^−^,CCR7^−^CD27^−^CD45RA^−^CD28^−^, and CCR7^−^CD27^−^CD45RA^+^CD28^−^, respectively (26–28). In accordance with our previous studies (5, 6), lung and tumor T cells did not express CCR7 (Supplementary Figure 2A). As such, there were barely any undifferentiated naïve (CD45RA^+^CD27^+^CD28^+^) T cells in the lung or tumor (Figures 2A–D). In the lung, CD103^+^ T_RM_ harbored mainly late differentiated CD28^−^CD45RA^−^CD27^−^ cells for both CD4^+^ and CD8^+^ lineages (Figures 2C,D; Supplementary Figure 2B). On the other hand, large fractions (40–50%) of lung CD69^+^ T_RM_ were early or intermediate differentiated. The differentiation profile of lung CD69^−^ T cells was more variable but mainly comprised of intermediate to late differentiated cells. Compared to lung T cell subsets, all TIL subsets contained less differentiated cells (Figures 2C,D). The largest differences were observed for the CD4^+^ TILs. CD103^+^CD4^+^ TILs contained more CD27^+^CD45RA^−^CD28^+^ early differentiated cells, while these cells were virtually absent in CD103^+^CD4^+^ T_RM_. This pattern was even more pronounced for the CD69^+^CD4^+^ and CD69^−^CD4^+^ subsets. CD103^+^CD8^+^ TILs had higher expression of CD27 than lung CD103^+^CD8^+^ T_RM_. In line with the CD4^+^ TILs, the strongest decrease in late differentiated cells was observed in the CD69^+^CD8^+^ and CD69^−^CD8^+^ TIL compartments. Of note, we also did not find differences in the phenotype of the T_RM_ or TILs between adenocarcinoma and squamous carcinoma (Supplementary Figures 2C,D). In summary, both CD4^+^ and CD8^+^ TILs, regardless of phenotype, contained less late differentiated cells compared to their lung equivalents.

### CD103^+^ TILs express common T_RM_ homing and adhesion molecules

**Figure 3:**
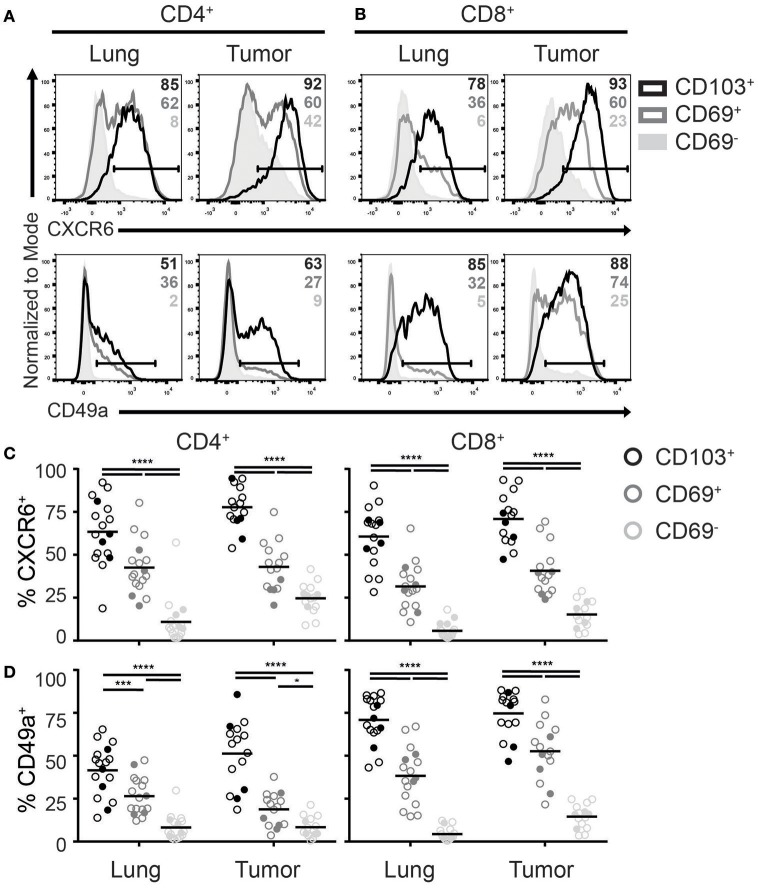
Expression of T_RM_ homing molecules by TILs. **(A-D)** The expression of chemokine receptor CXCR6 and integrin CD49a were analyzed on CD4^+^ and CD8^+^ T_RM_ and TILs. The expression of CXCR6 (top panels) and CD49a (bottom panels) on lung (left panels) and tumor (right panels) CD4^+^ **(A)** and CD8^+^ **(B)** T cell subsets is shown by representative histogram overlays (maximum set to 100%) (CD103^+^ black, CD69^+^ dark gray, CD69^−^ solid light gray). The frequencies of CXCR6^+^ **(C)** and CD49a^+^ **(D)** CD103^+^ (black circles), CD69^+^ (dark gray circles), and CD69^−^ (light gray circles) cells of lung and tumor CD4^+^ T cells (left graphs) and CD8^+^ T cells (right graphs). **(C,D)** The quantifications are shown as dot plots with the horizontal line indicating the mean and each point represents a unique sample. *n* = 15–17. Open circles and solid circles indicate adeno- and squamous carcinoma, respectively. ^*^p < 0.05, ^***^p < 0.001, ^****^p < 0.0001; 2-way ANOVA with Tukey's multiple comparisons test.

Recently, homing and adhesion molecules CXCR6 and integrin CD49a (α subunit of α1β1 integrin), were found in numerous T_RM_ core signatures and promote formation and retention of T_RM_ (14, 17, 29). Although CD8^+^ TILs were previously demonstrated to express CXCR6 (21), it remains unclear if this chemokine receptor defines TILs with a T_RM_ phenotype in tumors. In line with the expression pattern in lungs, we found that CD4^+^ and CD8^+^ TILs with a T_RM_ phenotype were enriched for CXCR6^+^ cells (Figures 3A,C). While CXCR6 was uniformly expressed by almost all CD103^+^ TILs, roughly half of the CD69^+^ TILs also expressed this chemokine receptor. Also in tumors CXCR6 expression appeared to define T_RM_, as CD69^−^ TILs barely expressed CXCR6, comparable with lung CD69^−^ T cells. Similarly to the CXCR6 expression, expression of CD49a was highest in CD103^+^, intermediate in CD69^+^, and absent in CD69^−^ TILs (Figures 3B,D). This was the case for both CD4^+^ and CD8^+^ TILs, albeit the CD49a expression was more uniform on CD8^+^ cells.

### Shared expression of transcription factors by T_RM_ and TILs with a T_RM_-like phenotype

**Figure 4:**
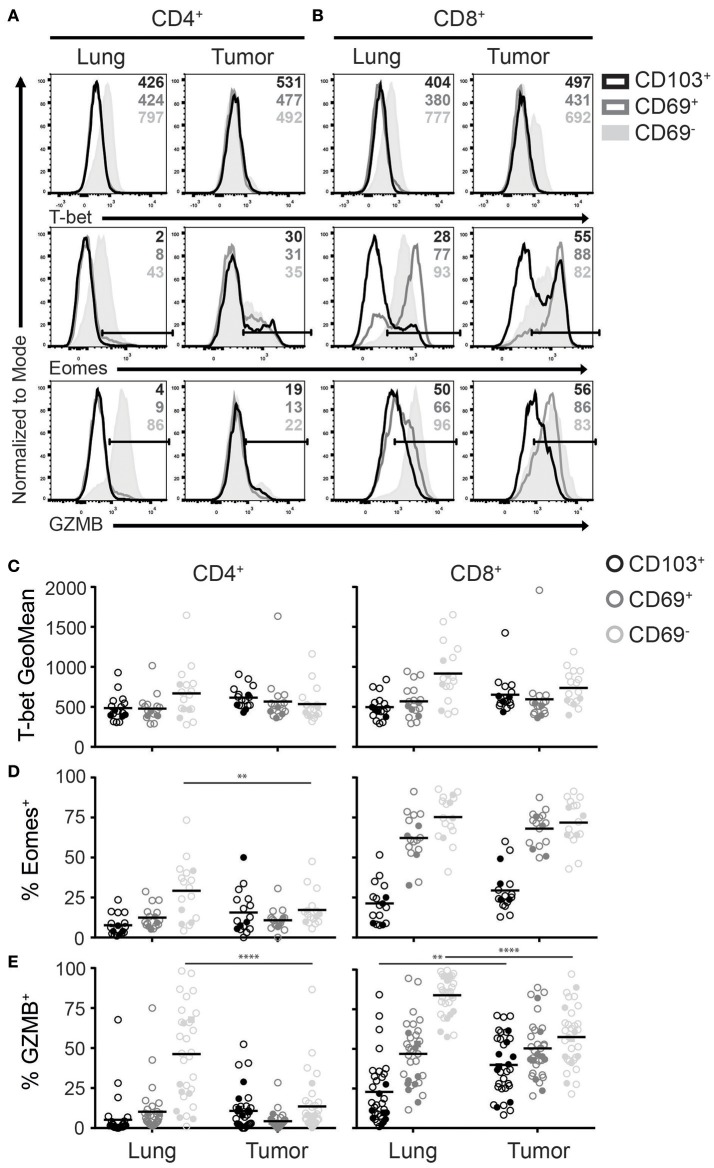
Expression of T-bet, Eomes and GZMB on T_RM_ and TILs. **(A–D)** The expression of T-bet, Eomes, and GZMB was analyzed on CD4^+^ and CD8^+^ T_RM_ and TILs. The expression of T-bet (top panels), Eomes (middle panels), and GZMB (bottom panels) on lung (left panels) and tumor (right panels) CD4^+^ **(A)** and CD8^+^ **(B)** T cells is shown by representative histogram overlays (maximum set to 100%) (CD103^+^ black, CD69^+^ dark gray, CD69^−^ solid light gray). The expression of T-bet (geometric mean fluorescence intensity; GeoMFI) **(C)** and frequencies of Eomes^+^ **(D)** and GZMB^+^ **(E)** CD103^+^ (black circles), CD69^+^ (dark gray circles), and CD69^−^ (light gray circles) cells of lung and tumor CD4^+^ (left graphs) and CD8^+^ (right graphs) T cells. **(C–E)** The quantifications are shown as dot plots with the horizontal line indicating the mean and each point represents a unique sample. *n* = 17. Open circles and solid circles indicate adeno- and squamous carcinoma, respectively. ^**^p < 0.01, ^****^p < 0.0001; 2-way ANOVA with Tukey's multiple comparisons test.

In both human and mice, T_RM_ express a different repertoire of transcription factors when compared to other memory and effector T cells (5, 6, 30). Among the most differentially expressed transcription factors are T-bet and Eomes. Downregulation of both T-box transcription factors is required T_RM_ development (31). Accordingly, lung CD103^+^CD4^+^, CD69^+^CD4^+^, and CD103^+^CD8^+^ T_RM_ expressed low levels of T-bet and Eomes (Figures 4A–D). In contrast, a substantial population of CD69^+^CD8^+^ T_RM_ expressed Eomes, while Tbet expression was similar to that of the other T_RM_ subsets. Of note, while T-bet expression was lower than in blood effector T cells, it was higher than blood-derived naïve T cells (Supplementary Figure 3A). Lung CD69^−^CD4^+^ and CD69^−^CD8^+^ subsets expressed the highest levels of T-bet and Eomes, similar to blood effector T cells (Figures 4A–D, Supplementary Figure 3A). TILs with a T_RM_ phenotype demonstrated comparable T-bet and Eomes expression patterns as their T_RM_ counterparts. However, Eomes expression was decreased in CD69^−^CD4^+^ TILs compared to lung CD69^−^CD4^+^ T cells, which fits with the decreased number of late differentiated cells in this subset, observed above. In line with the less differentiated phenotype of the TILs and the requirement of T_RM_ to downregulate T-box transcription factors, we determined whether TILs expressing CD27 also expressed T-bet and Eomes. Interestingly, CD8^+^ TILs that expressed CD27 also expressed Eomes. However, we did not find this pattern for CD4^+^ TILs, suggesting that there is correlation between the downregulation of CD27 and Eomes in CD8^+^ TILs but not in CD4^+^ TILs (Supplementary Figure 3B). We also determined Foxp3 expression in CD4^+^ TILs with a T_RM_ phenotype and found that most regulatory T cells (T_reg_) were found in the CD4^+^CD69^+^ TIL compartment (Supplementary Figure 3C).

### Granzyme B (GZMB) expression by T_RM_ and TILs

Since both T-bet and Eomes are important for effector cell differentiation and function (32), we determined granzyme B (GZMB) expression among the different T cell subsets. In line with the observed T-bet and Eomes expression, the frequency of GZMB^+^ cells was highest in lung CD69^−^ T cells (Figures 4A,E). However, there was a strong decrease of GZMB expression in CD69^−^ TILs compared to lung T cells. As for the CD69^+^ TIL subsets, GZMB expression resembled the levels of their lung counterparts. Most CD103^+^CD4^+^ and CD103^+^CD8^+^ lung T_RM_ lacked expression of GZMB, yet there was a significant increase of GZMB^+^ cells in CD103^+^CD8^+^ TILs. Overall, GZMB expression patterns were similar to those of T-bet and Eomes in both lung T_RM_ and TILs.

### CD103^+^ TILs expressed the highest levels of inhibitory receptors

**Figure 5:**
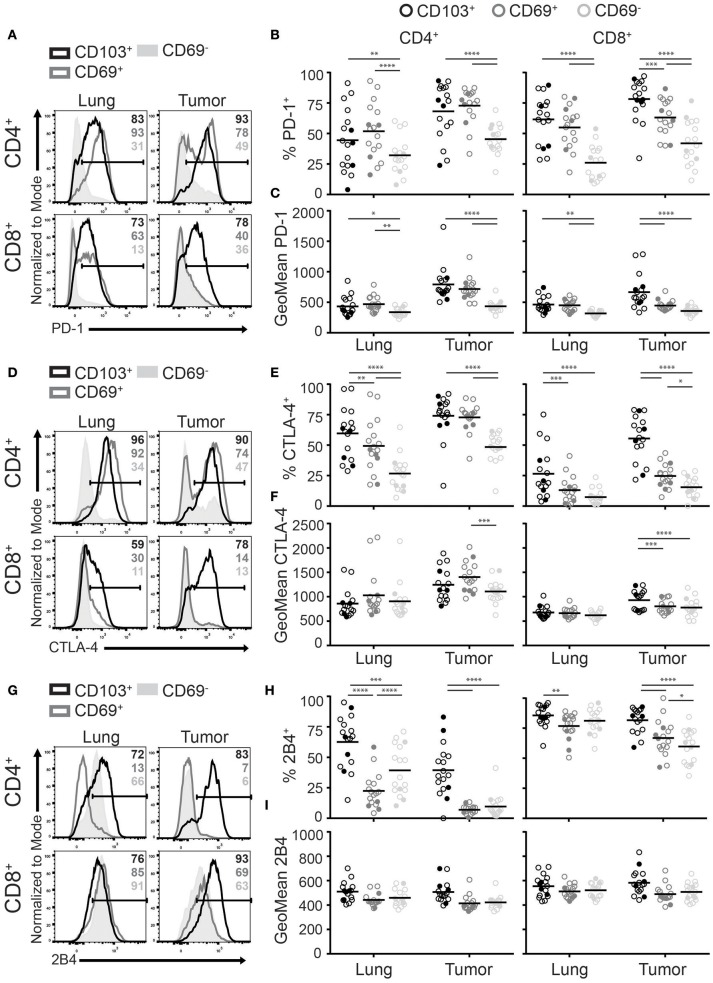
CD103^+^ TILs express the highest levels of inhibitory receptors. **(A–I)** The expression of inhibitory receptors PD-1, CTLA-4, and 2B4 was analyzed on CD4^+^ and CD8^+^ T_RM_ and TILs. The expression of PD-1 **(A)**, CTLA-4 **(D)**, and 2B4 **(G)** on lung (left panel) and tumor (right panel) on CD4^+^ (top panel) and CD8^+^ (bottom panel) T cells is shown by representative histogram overlays (maximum set to 100%) (CD103^+^ black, CD69^+^ dark gray, CD69^−^ solid light gray). The frequencies and geoMFI of PD-1^+^ **(B,C)**, CTLA-4^+^ **(E,F)** and 2B4^+^ **(H,I)** were quantified for CD103^+^ (black circles), CD69^+^ (dark gray circles), and CD69^−^ (light gray circles) cells of lung and tumor CD4^+^ (left graphs) and CD8^+^ (right graphs) T cells. **(B,C,E,F,H,I)** The quantifications are shown as dot plots with the horizontal line indicating the mean and each point represents a unique sample. *n* = 17. Open circles and solid circles indicate adeno- and squamous carcinoma, respectively. ^*^p < 0.05, ^**^p < 0.01, ^***^p < 0.001, ^****^p < 0.0001; 2-way ANOVA with Tukey's multiple comparisons test.

A shared feature of T_RM_ in mice and human is the expression of multiple inhibitory receptors (30). These receptors are thought to help protect against excessive T_RM_ activation and subsequent immunopathology of delicate tissues. In the tumor environment, upregulation of inhibitory receptors, such as PD-1, have also been linked to exhaustion (33). Paradoxically, PD-1 expression has also been described as a favorable prognostic marker in several cancers, in which it defines tumor-specific CD8^+^ T cells (34, 35). As several inhibitory molecules are targeted by immunotherapy, we investigated the expression of PD-1, CTLA-4, and 2B4 among the different TIL populations. PD-1 was broadly expressed by CD4^+^ and CD8^+^ T_RM_ and TILs (Figure 5A). The highest frequencies and levels of PD-1 expression were found on CD103^+^CD4^+^, CD69^+^CD4^+^, and CD103^+^CD8^+^ TILs followed by CD69^+^CD8^+^ TILs (Figures 5B,C). We found that the expression pattern of CTLA-4 was comparable to that of PD-1 (Figures 5D–F). Interestingly, CD4^+^ TILs expressed higher CTLA-4 levels than their CD8^+^ counterparts. Expression of 2B4 appeared different. While 2B4 is associated with T cell exhaustion, it functions differently from classical inhibitory receptors and can also act as a co-stimulatory molecule (33, 36). Though virtually all lung CD8^+^ T cells were 2B4^+^, 2B4 was also expressed by most CD103^+^CD4^+^ T_RM_ and some CD69^−^CD4^+^ T cells. In the tumor, frequencies of 2B4^+^ cells were again highest on CD103^+^ TILs. In comparison to the lung, CD69^+^ and CD69^−^ TILs expressed low or decreased 2B4 levels for the CD4^+^ and CD8^+^ populations, respectively (Figures 5G,H). Expression levels of 2B4 were comparable between the lung and tumor (Figure 5I). Overall, CD103^+^ TILs expressed the most inhibitory receptors.

### CD103^+^CD4^+^ TILs are the most potent cytokine producers in tumors

**Figure 6:**
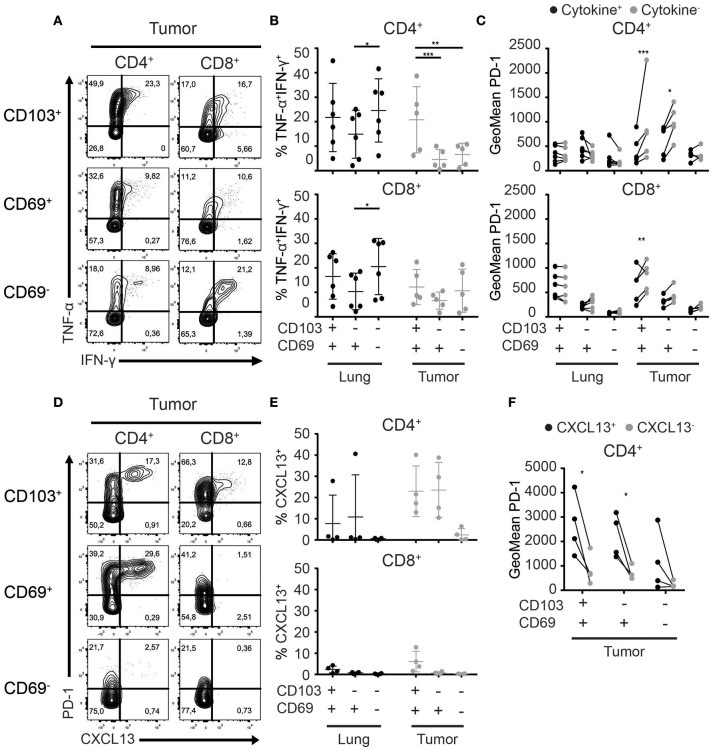
Cytokine and chemokine production of T_RM_ and TILs. **(A–C)** Cytokine production by lung T_RM_ and TILs was determined after overnight αCD3/αCD28 stimulation. **(A)** The production of TNF-α and IFN-γ by CD103^+^ (top panels), CD69^+^ (middle panels), and CD69^−^ (bottom panels) tumor CD4^+^ (left panels) and CD8^+^ (right panels) TILs shown by representative contour plots (TNF-α on y-axis, IFN-γ on x-axis). **(B)** TNF-α^+^IFN-γ^+^ CD103^+^, CD69^+^, and CD69^−^ cells of lung and tumor CD4^+^ (top graph) and CD8^+^ (bottom graph) T cells. **(C)** PD-1 expression (geometric mean fluorescence intensity; GeoMFI) was quantified on cytokine^+^ (TNF-α^+^ and/or IFN-γ^+^) (black circles) and cytokine^−^ (TNF-α^−^IFN-γ^−^) (gray circles) CD103^+^, CD69^+^, and CD69^−^ lung and tumor CD4^+^ (top graph) and CD8^+^ (bottom graph) T cells. **(D)** The expression of CXCL13 was determined by flow cytometry in CD103^+^ (top panels), CD69^+^ (middle panels), and CD69^−^ (bottom panels) tumor CD4^+^ (left panels) and CD8^+^ (right panels) TILs and is shown by representative contour plots (PD-1 on y-axis, CXCL13 on x-axis). **(E)** CXCL13^+^ of CD103^+^, CD69^+^, and CD69^−^ cells was quantified in lung and tumor CD4^+^ (top graph) and CD8^+^ (bottom graph) T cells. **(F)** PD-1 expression (GeoMFI) was quantified on the CXCL13^+^ (black circles) and CXCL13^−^ (gray circles) CD103^+^, CD69^+^, and CD69^−^ CD4^+^ TILs. *n* = 4–6 paired lung-tumor samples; all adenocarcinoma. ^*^p < 0.05, ^**^p < 0.01, ^***^p < 0.001; 2-way ANOVA with Tukey's multiple comparisons test.

The main obstacle faced by TILs is exhaustion induced by repeated stimulation and subsequent loss of T cell receptor responsiveness. A common feature of exhausted T cells is a step-wise loss of the capacity to produce multiple cytokines upon activation (37, 38). Thus, we set out to test the functionality of the different CD4^+^ and CD8^+^ T_RM_ and TIL subsets. We stimulated T_reg_-depleted T cells with plate-bound agonistic αCD3 and soluble αCD28 antibodies and determined cytokine production. The majority of CD4^+^ T_RM_ and TILs upregulated CD40L and/or CD137 upon activation. CD8^+^ T_RM_ and TIL activation was restricted to CD137 upregulation (Supplementary Figures 4A,C). In terms of cytokine production, CD103^+^CD4^+^ TILs produced significantly more TNF-α and IFN-γ than CD69^+^CD4^+^ and CD69^−^CD4^+^ TILs (Figures 6A,B; Supplementary Figures 4B,C). No differences in cytokine production were observed between CD103^+^CD8^+^ and CD69^+^CD8^+^ TIL and T_RM_ fractions. Cytokine production of CD103^+^CD4^+^ TILs also exceeded that of all CD8^+^ TIL populations.

### PD-1 expression delineates between functionally distinct subsets of CD4^+^ TILs

As we demonstrated the expression of PD-1 to be highest on TILs with a T_RM_ phenotype and CD103^+^CD4^+^ TILs to be the best cytokine producers, we investigated the relationship between PD-1 expression and cytokine production. To do so, we determined the expression of PD-1 (geometric mean fluorescence intensity) on T_RM_ and TILs that produced cytokines (positive for TNF-α and/or IFN-γ) and TILs that did not produce cytokines (TNF-α^−^IFN-γ^−^; Figure 6C; Supplementary Figure 4D). For lung CD4^+^ and CD8^+^ T_RM_, there was no differential expression of PD-1 between the cytokine producing or non-producing cells. On the other hand, within CD103^+^CD4^+^, CD69^+^CD4^+^, and CD103^+^CD8^+^ TIL populations, significantly lower expression of PD-1 was observed for the cytokine producers. Recently, PD-1^++^CD4^+^ TILs in breast cancer and PD-1^++^ CD8^+^ TILs in NSCLC were shown to produce CXCL13 (39, 40). Therefore, we determined CXCL13 expression in the different T_RM_ and TIL subsets. Also in the CD4^+^ T cells, expression of CXCL13 appeared to be biased to the tumor fraction. A high percentage of CD4^+^ TILs expressed CXCL13 in 4 out of 4 tested samples while only in 1 out of 4 lung samples expression was detected. Strikingly CXCL13 was only expressed by TIL with a T_RM_ phenotype. The highest numbers of CXCL13^+^ cells were detected in the CD4^+^ lineage (Figures 6D,E). Furthermore, CXCL13 was solely expressed by PD-1^++^ TILs (Figures 6D,F). Thus, we found that PD-1 expression defines functionally distinct subsets of CD4^+^ TILs, effector cytokine producer PD-1^low^ and CXCL13 producing PD-1^++^ TILs.

### Co-stimulation increases cytokine production of TILs

**Figure 7:**
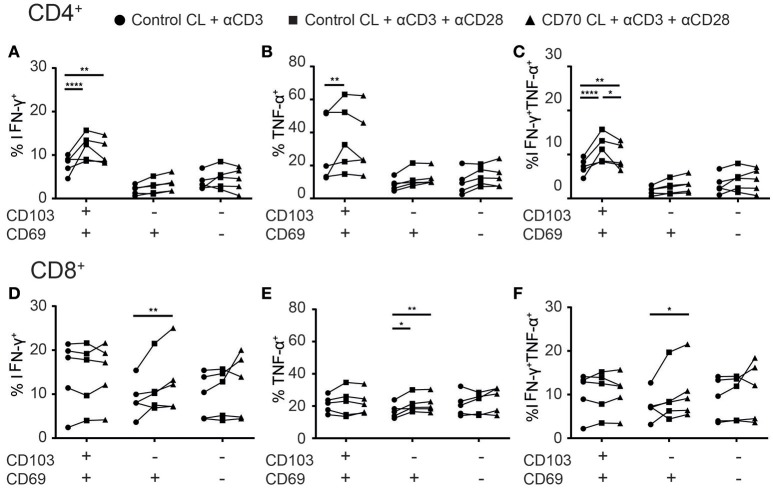
Co-stimulation enhances cytokine production of TILs. **(A–F)** The effect of co-stimulation on cytokine production of TILs was assessed with overnight stimulation. **(A–C)** The frequencies of IFN-γ^+^ **(A)**, TNFα^+^ **(B)**, or TNF-α^+^IFN-γ^+^ **(C)** CD103^+^, CD69^+^, and CD69^−^ CD4^+^ TILs stimulated with αCD3 with control cell line (CL) (black circles), αCD3 and αCD28 with control CL (black squares), or αCD3 and αCD28 with CD70-expressing CL (black triangles) was quantified. **(D–F)** The frequencies of IFN-γ^+^ **(D)**, TNFα^+^ **(E)**, or TNF-α^+^IFN-γ^+^ **(F)** CD103^+^, CD69^+^, and CD69^−^ CD8^+^ TILs stimulated with αCD3 with control CL (black circles), αCD3 and αCD28 with control CL (black squares), or αCD3 and αCD28 with CD70-expressing CL (black triangles) was quantified. *n* = 5. ^*^p < 0.05, ^**^p < 0.01, ^****^p < 0.0001; 2-way ANOVA with Tukey's multiple comparisons test.

Adoptive transfer and vaccination strategies to treat cancer have demonstrated that CD4^+^ T cell help, through co-stimulation, is required for optimal cytotoxic CD8^+^ T cell responses in tumors. Administration of co-stimulation in combination with PD-1 therapy improved the cytokine production of TILs in tumor-bearing mice (22). We next assessed whether CD28 and CD27 co-stimulation in addition to TCR triggering could boost cytokine production of T_reg_-depleted TILs. CD103^+^CD4^+^ TILs, but not other CD4^+^ TILs, mainly responded to CD28 co-stimulation by producing more IFN-γ and/or TNF-α (Figures 7A–C). However, agonistic stimulation of CD27 did not add to this, which could be explained by higher CD28 than CD27 expression by the CD103^+^CD4^+^ TILs. While CD103^+^CD8^+^ TILs appeared non-responsive to co-stimulation, agonistic CD28 stimulation boosted TNF-α production by CD69^+^CD8^+^ TILs (Figure 7E). The addition of CD27 co-stimulation further enhanced TNF-α and/or IFN-γ production (Figures 7D–F). We did not find differences between adenocarcinoma and squamous carcinoma samples (Supplementary Figure 5). These data suggest that therapeutic efficacy of cancer immunotherapy targeting specific TIL populations may improve by providing agonistic stimulation of co-stimulatory molecules.

## Discussion

In this study, we investigated the phenotype of tumor infiltrating T cells in NSCLC. We phenotypically characterized CD4^+^ and CD8^+^ TILs and directly compared these with T cell populations in the surrounding lung tissue. While adaptive immune responses that protect against tumors are typically attributed to CD8^+^ T cells, several studies provide evidence that CD4^+^ T cells also play a central role (41). As CD4^+^ T cells exhibit phenotypic and functional heterogeneity, different subsets are expected to play different and even opposing roles in the tumor environment. While accumulation of CD4^+^ T_reg_ within tumors is associated with worse prognoses in many cancers (42), CD4^+^ T helper cells are required to optimize cytotoxic CD8^+^ T cell responses against tumor cells (43). In addition, CD4^+^ T cells were demonstrated to mediate tumor-antigen-mediated killing of tumor cells, highlighting the importance to understand the functional heterogeneity of the different T cell subsets in tumors (24, 44, 45).

While numerous studies have reported the presence of T_RM_-like CD8^+^ T cells in solid tumors to be a favorable prognosis, the role of CD4^+^ TILs with a shared phenotype is unclear. We demonstrated for the first time a positive correlation between CD103^+^CD8^+^ and CD103^+^CD4^+^ TILs in NSCLC. Our findings are supported by the observation that CD8^+^ TILs from CD103-rich tumors expressed transcripts linked to CD4^+^ T cell-mediated help, while CD8^+^ TILs from CD103-poor tumors did not (21). As CD4^+^ T cell help was demonstrated to be required for guiding CD8^+^ T_RM_ formation in the lungs by regulating the entry of T_RM_ precursors to the lung mucosa (46), it is tempting to speculate that a similar role applies in NSCLC. A key mechanism to attract T_RM_ precursors into the tissue is IFN-γ production by CD4^+^ T cells (46, 47). IFN-γ induces the production of chemokines by the tissue and boosts the expression of adhesion molecules by the vasculature which result in higher T cell infiltration (7, 8). In the tumor, we found the best producers of IFN-γ to be CD103^+^CD4^+^ TILs. While adapted to the metabolic requirements in tissues, such specialization may also provide CD103^+^CD4^+^ T cells with an advantage over other CD4^+^ T cell subsets in malignant niches. Strategies designed to boost anti-tumor CD8^+^ CTL responses may therefore benefit from taking into account the CD4^+^ subset that appears most effective for their generation.

Once in the tissue, CD8^+^ T_RM_ maturation is believed to be independent of CD4^+^ T cell help. T_RM_ maturation is driven by local inflammatory stimuli that induce the expression of CD69 and CD103 (48). In the healthy tissue many of these signals are provided by local macrophages and dendritic cells, which were demonstrated to be crucial for full maturation of especially CD4^+^ T_RM_ (49–51). It remains to be investigated if the increased frequency of phenotypically less-differentiated T_RM_-like cells in NSCLC may be the result of the tumor environment that suppresses dendritic cell function (52). Full maturation of CD103^+^ T_RM_ requires TGF-β signaling (31). Several lung tumors are described to express high levels of TGF-β (53), which may explain the high level of CD103^+^CD8^+^ TILs in NSCLC. At apparent odds, we found the frequencies of CD103^+^CD4^+^ TILs to be decreased relative to the surrounding lung tissue. The altered ratio between CD103^+^CD8^+^ and CD103^+^CD4^+^ TILs in NSCLC may be a result of different requirements for their maintenance. While CD8^+^ T_RM_ maintenance was described to be independent of persistent antigen (54), whether antigen presence is required for the maintenance of CD103^+^CD4^+^ T_RM_ remains unclear. If CD103^+^CD4^+^ T_RM_ maintenance is antigen-dependent, this may be a major hurdle for CD103^+^CD4^+^ TILs as many tumors express little or no MHC class II molecules (55). Strikingly, tumor cells upregulate MHC class Il molecules and consequently their cytotoxicity in response to IFN-γ. Moreover, adoptive transfer of Th1-like CD4^+^ T cells was found to protect against tumors lacking MHC class II expression (24, 44, 56). Our data suggest the CD103^+^CD4^+^ TILs to be the best candidates for such therapies.

In NSCLC, IFN-γ-responsive gene expression signatures are associated with favorable prognosis (57). In light of our findings, a prominent role for CD103^+^CD4^+^ TILs seems possible as they were the most potent intratumoral cytokine producing T cell subset, despite the high expression of PD-1 and CTLA-4. These CD103^+^CD4^+^ TILs also expressed 2B4 while other CD4^+^ TILs did not. While generally 2B4 is considered an inhibitory receptor, it functions differently from typical inhibitory receptors and has also been demonstrated to act as a co-stimulatory molecule depending on the availability of intracellular SAP protein (58). Therefore, 2B4 may be playing a different role on CD103^+^CD4^+^ TILs. On the other hand, we found that PD-1 expression delineates between effector cytokine and CXCL13 producing CD4^+^ TILs. TILs with high PD-1 expression are classically thought to be exhausted since they do not produce effector cytokines. This raised the question of how PD-1^++^CXCL13^+^ cells act in the tumor environment. Our data suggests that CXCL13^+^ TILs are functionally adapted to the tumor environment rather than being exhausted. As a mechanism, intratumoral CXCL13 production may serve to recruit CXCR5^+^ T follicular helper cells (Tfh) or B cells. Recently, it was shown that PD-1^++^CD8^+^ TILs are localized within tertiary lymphoid structures (TLS) in tumors and may be important for the formation of TLS (40). As such, PD-1 has also been shown to control the positioning and function of Tfh, which also produce CXCL13 (39, 59). Therefore, these PD-1^++^CD4^+^ TILs may also be located within TLS and contribute to the formation of TLS in tumors of NSCLC. It has been shown that in chronic viral infections, a subset of memory CD8^+^ T cells with an “exhausted” phenotype retain their effector function through TCF-1 (60), indicating that these phenotypically exhausted T cells contain diverse subsets. Our data reveal the functional heterogeneity within these “exhausted” CD4^+^ TILs, suggesting that not all of these TILs are exhausted but functionally distinct from the effector cytokine producing TILs.

Agonistic activation of co-stimulatory CD27 and CD28 boosted cytokine production of the CD103^+^CD4^+^ TILs. However, CD69^+^CD4^+^ TILs expressed identical levels of PD-1 and higher levels of CD27 and CD28, but cytokine production was not boosted with additional co-stimulation. If parallels may be drawn with differentiation of circulating T cells associated with a step-wise loss of CD27 and CD28, our data suggests these CD69^+^CD4^+^ cells are less differentiated and adapted to the tissue niche. On the other hand, while CD69^−^CD4^+^ TILs have lower expression of inhibitory receptors, this subset was not able to produce effector cytokines to the same extent as CD103^+^CD4^+^ TILs. However, CD69^−^CD4^+^ TILs mainly consists of early and early-like differentiated cells, which could indicate that these cells are recent emigrants and are yet to fully differentiate. This is also supported by the lower expression of co-inhibitory molecules which suggest they are not yet exhausted by the tumor microenvironment. Recently, it has been suggested that these phenotypically “exhausted” TILs are in a stage of differentiation rather than exhausted and that this state of “exhaustion” may be reversible (61–63). Overall, both CD4^+^ and CD8^+^ TILs expressed high levels of CD27 and CD28, suggestive of cells in an early stage of differentiation. Perhaps the addition of co-stimulation to current cancer vaccines and immunotherapies could push the differentiation of TILs into optimal cytotoxic effector cells and enhance the efficacy of cancer therapies.

While IFN-γ production may directly inhibit tumor growth in synergy with TNF-α (64, 65), it remains to be investigated whether CD103^+^CD4^+^ TILs are equally equipped to kill cancer cells as their CD103^+^CD8^+^ counterparts (19). CD49a expression by CD103^+^CD4^+^ and CD103^+^CD8^+^ TILs may allude to this, as CD49a expressing CD103^+^CD8^+^ TILs were the most potent killers of tumor cells in a mouse model of melanoma and CD49a defines cytotoxic CD8^+^ T_RM_ in skin (16, 66, 67). As such, strategies to identify CD4^+^ T cells that can directly target tumor cells may focus on CD103^+^CD4^+^ T cells. Therapeutic manipulation of such reactivity could be a highly attractive strategy.

## Materials and methods

### Subjects

Lung and tumor tissue samples were obtained from a total of 33 non-small cell carcinoma (NSCLC) patients. The patients received a surgical resection of primary tumors as first line therapy without prior chemo- or radiotherapy. Blood was drawn from a central line at the start of surgery. Patients included were stages AJCC between IA1 and IIIA. The exclusion criteria included history of asthma or a recent lower respiratory tract infection. The patients were recruited from Onze Lieve Vrouwe Gasthuis (OLVG), Amsterdam, the Netherlands. A list of the age, gender, pathology of the patients used in this study are listed in Supplementary Table 1.

### Study approval

Written informed consent was given by all of the patients and donors before inclusion into the study. The Ethical Review Board (ERB) of the METC/CCMO of the OLVG approved the study under the MEC-U number NL52453.100.15 according to the Declaration of Helsinki.

### Isolation of mononuclear cells from peripheral blood and lung tissue

Peripheral blood mononuclear cells (PBMCs) were isolated from heparinized peripheral blood samples with standard density gradient techniques. For the lung material, after the lobectomy the pathologist cuts off a piece of peripheral normal looking lung tissue farthest away from the tumor. For the tumor material, the pathologist cuts off a piece of the tumor. Lung mononuclear cells (LMC) and tumor mononuclear cells (TMC) were isolated from the tissues as previously described (68, 69). In short, the tissue was cut into small pieces and incubated for 1 h at 37°C in digestion medium [RPMI with 20 mM Hepes, 10% fetal calf serum (FCS), 50 U/ml DNAse type I (Sigma-Aldrich), 300 U/ml collagenase type 4 (Worthington)] while shaking. Before and after the digestion, the tissue was dissociated using gentleMACS Tissue Dissociator (Miltenyi). The digested tissue was passed through a flow-through chamber to achieve a single cell suspension. To isolate mononuclear cells from the cell suspension standard density gradient techniques were used. LMC, TMC, and PBMC samples were cryopreserved in liquid nitrogen until further analysis.

### Flow cytometry analysis

PBMC or LMC were labeled with combinations of the following antibodies: anti-CD4, anti-CD3, anti-CD8, anti-CD27, anti-CD45RA, anti-CD69, anti-CD103, anti-CD49a, anti-CXCR6, anti-CD28, anti-CCR7, anti-PD-1, and anti-2B4. Near-IR fixable dye (Invitrogen) was used to exclude dead cells from the analysis. For intracellular staining the following antibodies were used: anti-CTLA4, anti-Eomes, anti-Tbet, anti-IFNγ, anti-GZMB, anti-CD40L, anti-CD137, anti-IL-2, anti-TNFα, and anti-CXCL13. The cells were labeled according to manufacturer's instructions. For the intracellular staining the cells with fixed and permeabilized using the Foxp3/Transcription Factor Staining kit (eBioscience). All samples were measured in PBS 0.5% FCS with a LSR Fortessa (BD) or FACSymphony (BD) and the analysis was performed using FlowJo Version 10 software. See Supplementary Table 2 for the full list of antibodies used in this manuscript.

### *In vitro* stimulation assays

Cytokine production by lung and tumor T cells was determined by incubating TMC with platebound αCD3 (HIT3A; eBioscience) and soluble αCD28 (s.28; CLB) overnight at 37°C in the presence of Brefeldin A (eBioscience). T_reg_ were depleted by MACS (Miltenyi) isolation CD25^+^ cells from the LMC and TMC samples before the stimulation according to manufacturer's protocol. To determine the effects of co-stimulation on TILs, TILs were incubated with only soluble αCD3 (HIT3A; eBioscience) with control cell line, soluble αCD3 and αCD28 (s.28; CLB) with control cell line, or soluble αCD3 (HIT3A; eBioscience) and αCD28 (s.28; CLB) with a CD70-expressing cell line. The cell lines were made by cloning CD70 cDNA into pMX-IRES-GFP vector using EcoRI and NotI restriction enzymes (NEB). Retroviral packaging by transfection of either pMX-IRES-GFP empty vector or pMX-hCD70-IRES-GFP together with pCL-ECO into Phoenix-ECO packaging cells using polyethyleminine. Supernatants containing retrovirus was collected 48 h after transfection and used for retroviral transduction of mouse NIH3T3 cells. Transduced NIH3T3 cells were sorted on GFPhigh (pMX-IRES-GFP) or GFPhighCD70high (pMX-hCD70-IRES-GFP) expression using a MoFlo Astrios cell sorter (Beckman Coulter).

### Statistics

To determine the significance of our results, we used 2-way ANOVA and Tukey's multiple comparisons test with GraphPad Prism 6. *p*-value of < 0.05 was considered statistically significant (^*^p < 0.05; ^**^p < 0.01; ^***^p < 0.001; ^****^p < 0.0001).

## Author contributions

AO and PH designed the project and experiments. All of the authors performed experiments and/or collected tissue and blood samples. All authors contributed to the interpretation and discussion of data. AO and PH wrote the manuscript. All authors read and approved the manuscript.

### Conflict of interest statement

The authors declare that the research was conducted in the absence of any commercial or financial relationships that could be construed as a potential conflict of interest.
