# Supplementary material for: Functional Heterogeneity of CD4+ Tumor-Infiltrating Lymphocytes With a Resident Memory Phenotype in NSCLC
Source: Front Immunol. 2018 Nov 16;9:2654. doi: 10.3389/fimmu.2018.02654 (PMC6250821; doi:10.3389/fimmu.2018.02654)
Supplement: Supplementary file 2 [file Presentation_1.PPTX]

## Slide 1
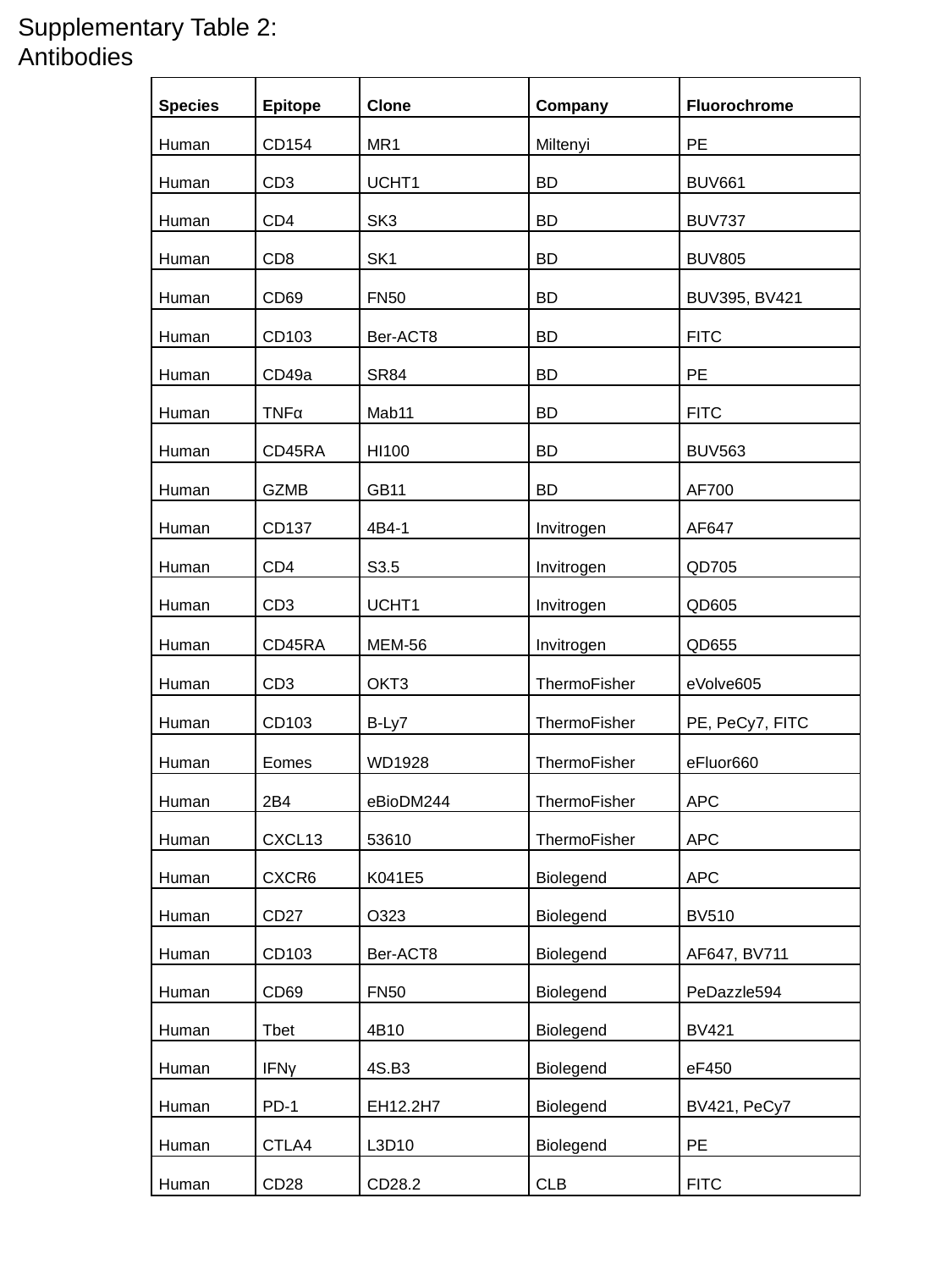

Supplementary Table 2:
Antibodies
| Species | Epitope | Clone | Company | Fluorochrome |
| --- | --- | --- | --- | --- |
| Human | CD154 | MR1 | Miltenyi | PE |
| Human | CD3 | UCHT1 | BD | BUV661 |
| Human | CD4 | SK3 | BD | BUV737 |
| Human | CD8 | SK1 | BD | BUV805 |
| Human | CD69 | FN50 | BD | BUV395, BV421 |
| Human | CD103 | Ber-ACT8 | BD | FITC |
| Human | CD49a | SR84 | BD | PE |
| Human | TNFα | Mab11 | BD | FITC |
| Human | CD45RA | HI100 | BD | BUV563 |
| Human | GZMB | GB11 | BD | AF700 |
| Human | CD137 | 4B4-1 | Invitrogen | AF647 |
| Human | CD4 | S3.5 | Invitrogen | QD705 |
| Human | CD3 | UCHT1 | Invitrogen | QD605 |
| Human | CD45RA | MEM-56 | Invitrogen | QD655 |
| Human | CD3 | OKT3 | ThermoFisher | eVolve605 |
| Human | CD103 | B-Ly7 | ThermoFisher | PE, PeCy7, FITC |
| Human | Eomes | WD1928 | ThermoFisher | eFluor660 |
| Human | 2B4 | eBioDM244 | ThermoFisher | APC |
| Human | CXCL13 | 53610 | ThermoFisher | APC |
| Human | CXCR6 | K041E5 | Biolegend | APC |
| Human | CD27 | O323 | Biolegend | BV510 |
| Human | CD103 | Ber-ACT8 | Biolegend | AF647, BV711 |
| Human | CD69 | FN50 | Biolegend | PeDazzle594 |
| Human | Tbet | 4B10 | Biolegend | BV421 |
| Human | IFNγ | 4S.B3 | Biolegend | eF450 |
| Human | PD-1 | EH12.2H7 | Biolegend | BV421, PeCy7 |
| Human | CTLA4 | L3D10 | Biolegend | PE |
| Human | CD28 | CD28.2 | CLB | FITC |

## Slide 2
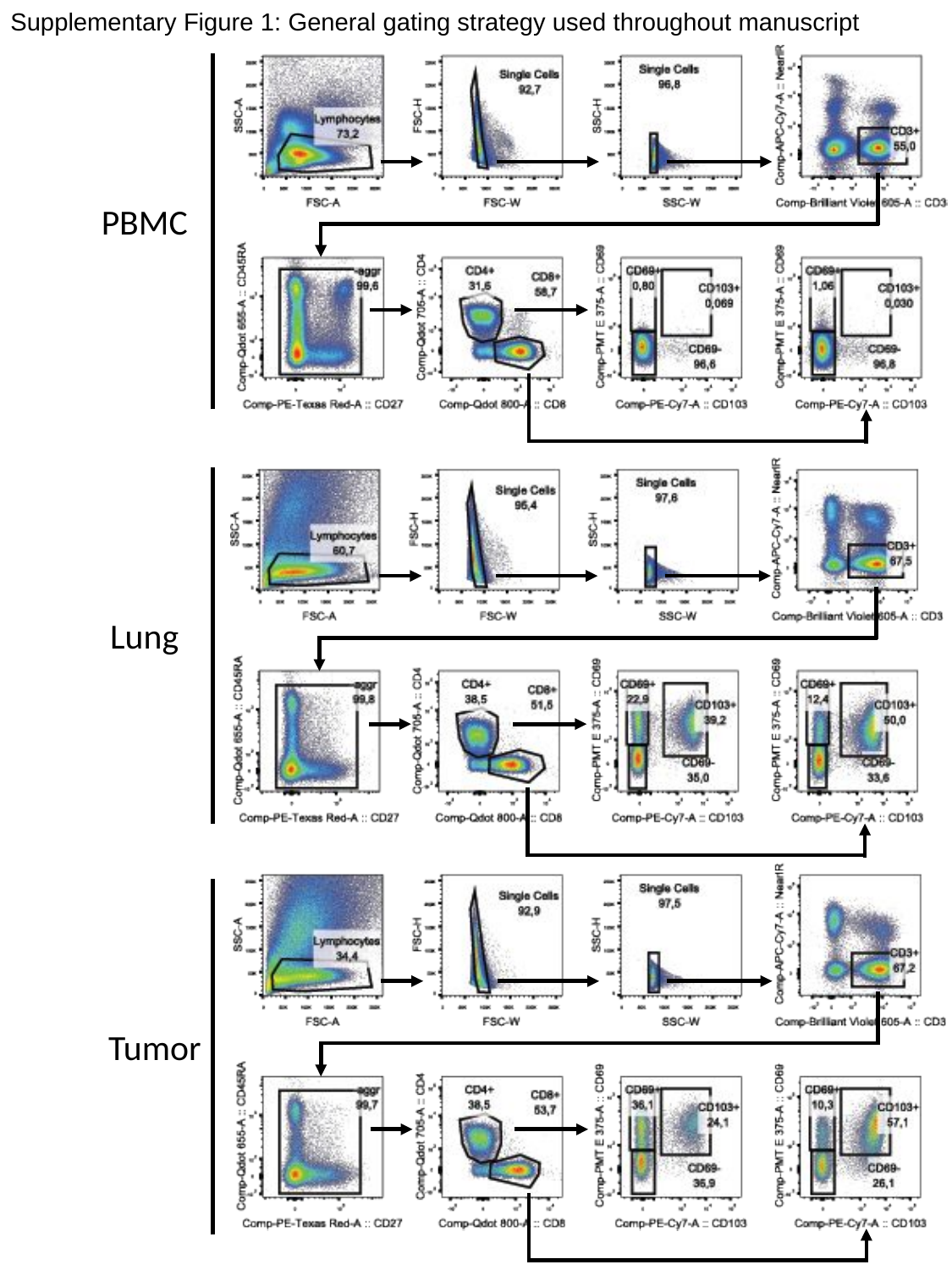

Supplementary Figure 1: General gating strategy used throughout manuscript
PBMC
Lung
Tumor

## Slide 3
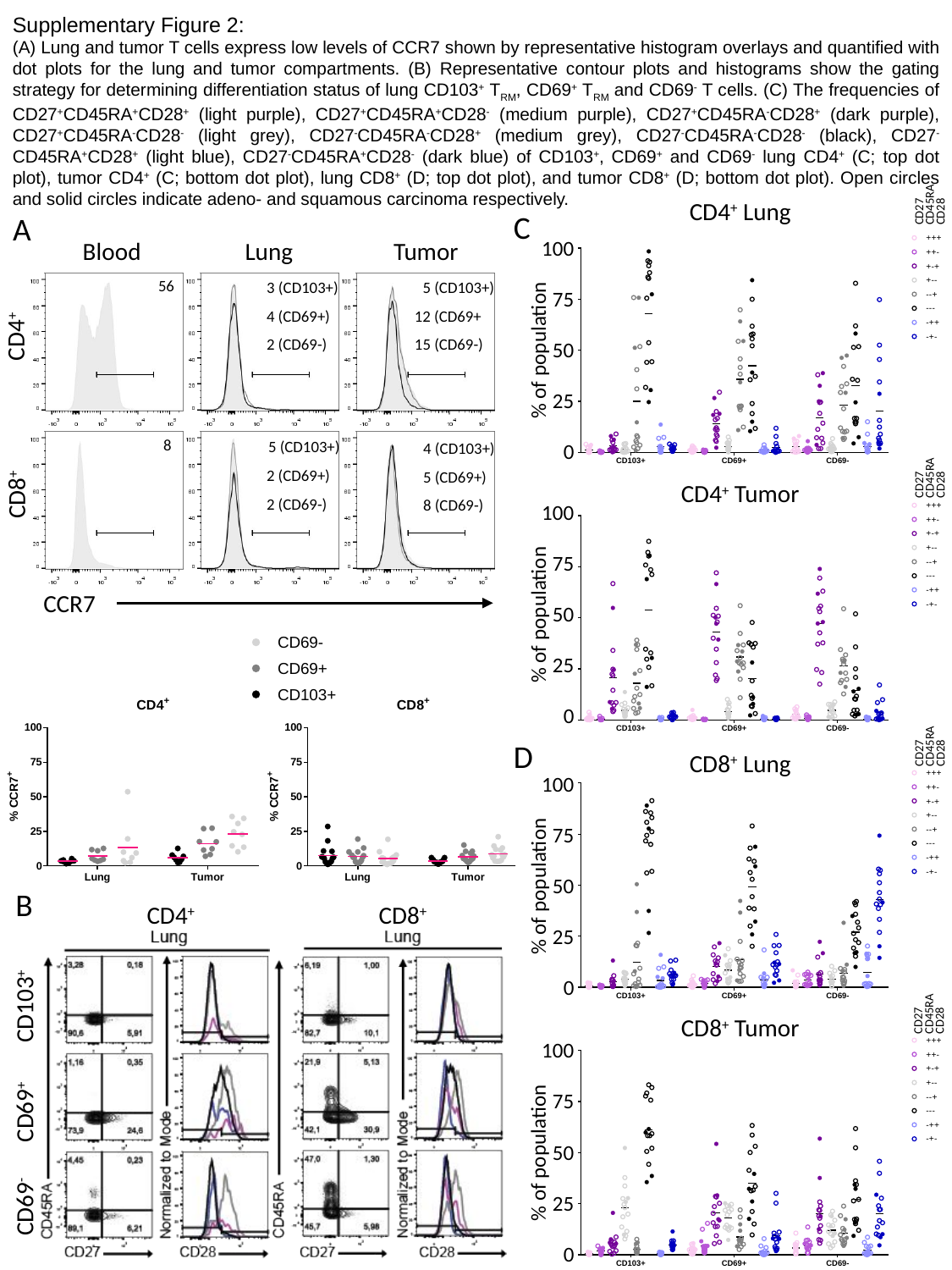

Supplementary Figure 2:
(A) Lung and tumor T cells express low levels of CCR7 shown by representative histogram overlays and quantified with dot plots for the lung and tumor compartments. (B) Representative contour plots and histograms show the gating strategy for determining differentiation status of lung CD103+ TRM, CD69+ TRM and CD69- T cells. (C) The frequencies of CD27+CD45RA+CD28+ (light purple), CD27+CD45RA+CD28- (medium purple), CD27+CD45RA-CD28+ (dark purple), CD27+CD45RA-CD28- (light grey), CD27-CD45RA-CD28+ (medium grey), CD27-CD45RA-CD28- (black), CD27-CD45RA+CD28+ (light blue), CD27-CD45RA+CD28- (dark blue) of CD103+, CD69+ and CD69- lung CD4+ (C; top dot plot), tumor CD4+ (C; bottom dot plot), lung CD8+ (D; top dot plot), and tumor CD8+ (D; bottom dot plot). Open circles and solid circles indicate adeno- and squamous carcinoma respectively.
CD4+ Lung
CD45RA
CD27
CD28
C
A
100
Blood
Lung
Tumor
56
3 (CD103+)
5 (CD103+)
75
4 (CD69+)
12 (CD69+
CD4+
2 (CD69-)
15 (CD69-)
50
% of population
25
8
5 (CD103+)
4 (CD103+)
0
2 (CD69+)
5 (CD69+)
CD45RA
CD27
CD28
CD4+ Tumor
CD8+
2 (CD69-)
8 (CD69-)
100
75
CCR7
50
% of population
25
0
D
CD45RA
CD27
CD28
CD8+ Lung
100
75
50
% of population
B
CD4+
CD8+
25
0
CD103+
CD45RA
CD8+ Tumor
CD27
CD28
100
75
CD69+
50
% of population
25
CD69-
0

## Slide 4
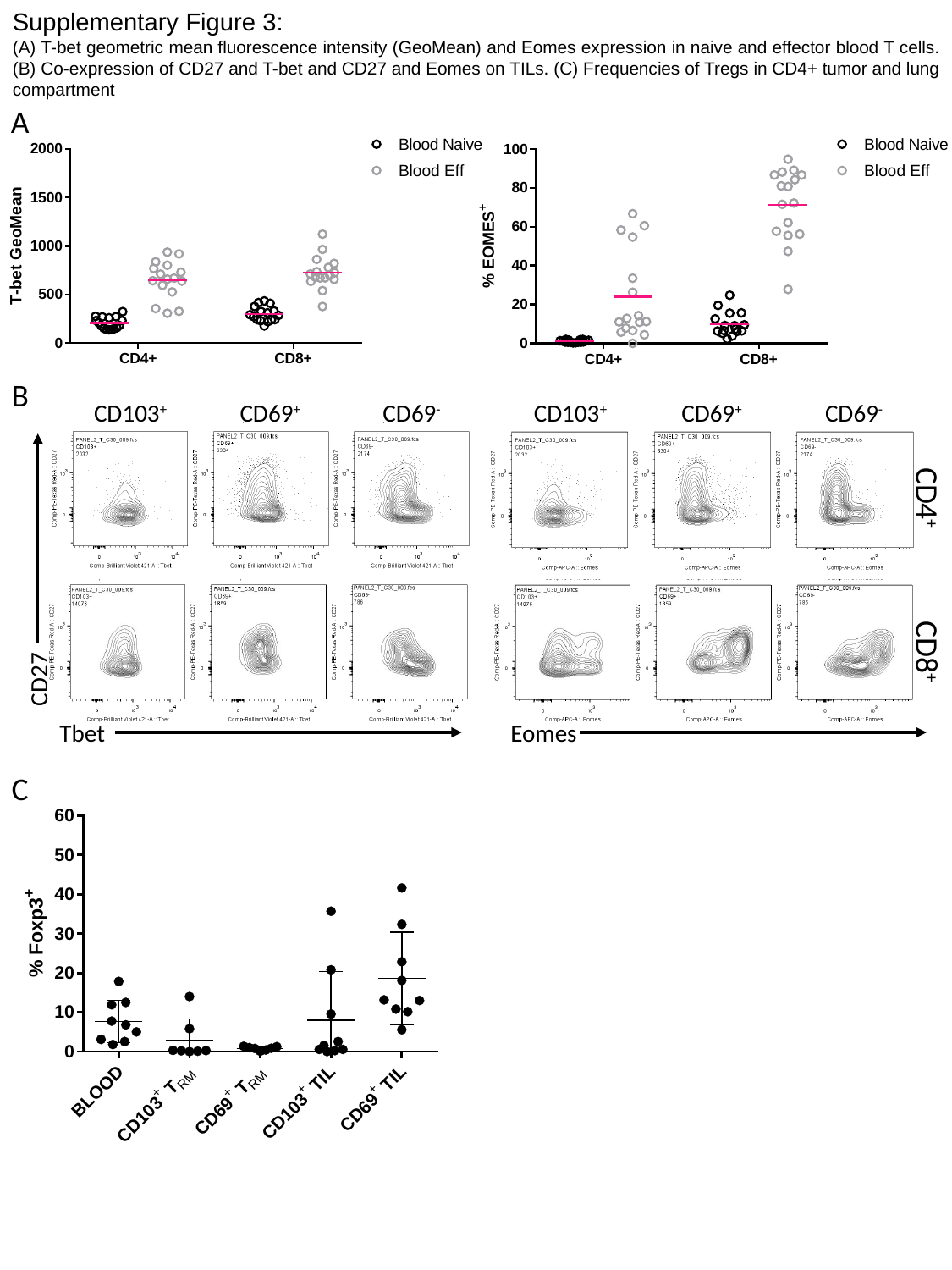

Supplementary Figure 3:
(A) T-bet geometric mean fluorescence intensity (GeoMean) and Eomes expression in naive and effector blood T cells. (B) Co-expression of CD27 and T-bet and CD27 and Eomes on TILs. (C) Frequencies of Tregs in CD4+ tumor and lung compartment
A
B
CD103+
CD69+
CD69-
CD103+
CD69+
CD69-
CD4+
CD8+
CD27
Tbet
Eomes
C

## Slide 5
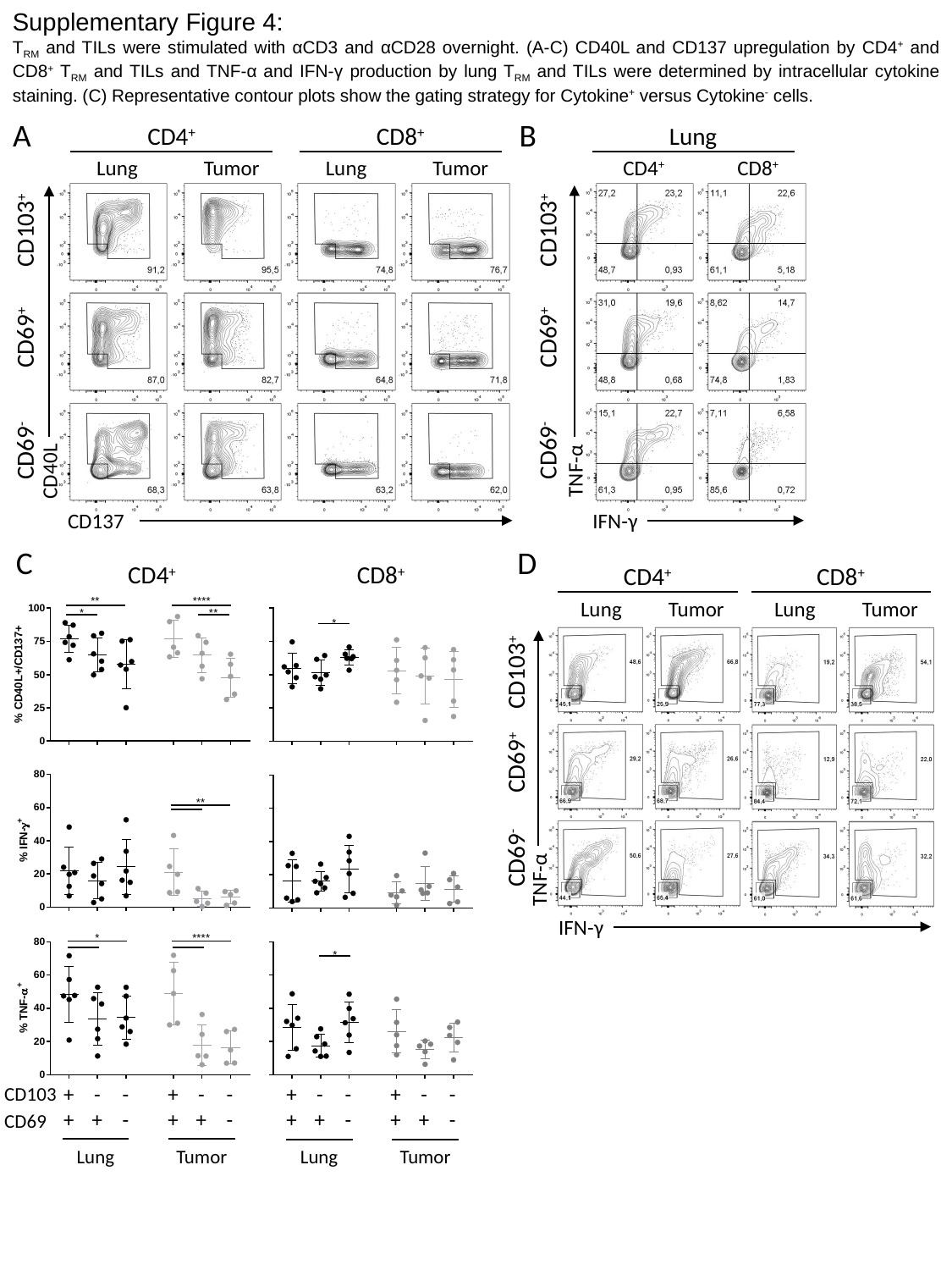

Supplementary Figure 4:
TRM and TILs were stimulated with αCD3 and αCD28 overnight. (A-C) CD40L and CD137 upregulation by CD4+ and CD8+ TRM and TILs and TNF-α and IFN-γ production by lung TRM and TILs were determined by intracellular cytokine staining. (C) Representative contour plots show the gating strategy for Cytokine+ versus Cytokine- cells.
A
B
CD4+
CD8+
Lung
Lung
Tumor
Lung
Tumor
CD4+
CD8+
CD103+
CD103+
CD69+
CD69+
CD69-
CD69-
TNF-α
CD40L
CD137
IFN-γ
C
D
CD4+
CD8+
CD4+
CD8+
Lung
Tumor
Lung
Tumor
CD103+
CD69+
CD69-
TNF-α
IFN-γ
+
+
-
+
-
-
+
+
-
+
-
-
+
+
-
+
-
-
+
+
-
+
-
-
CD103
CD69
Lung
Tumor
Lung
Tumor

## Slide 6
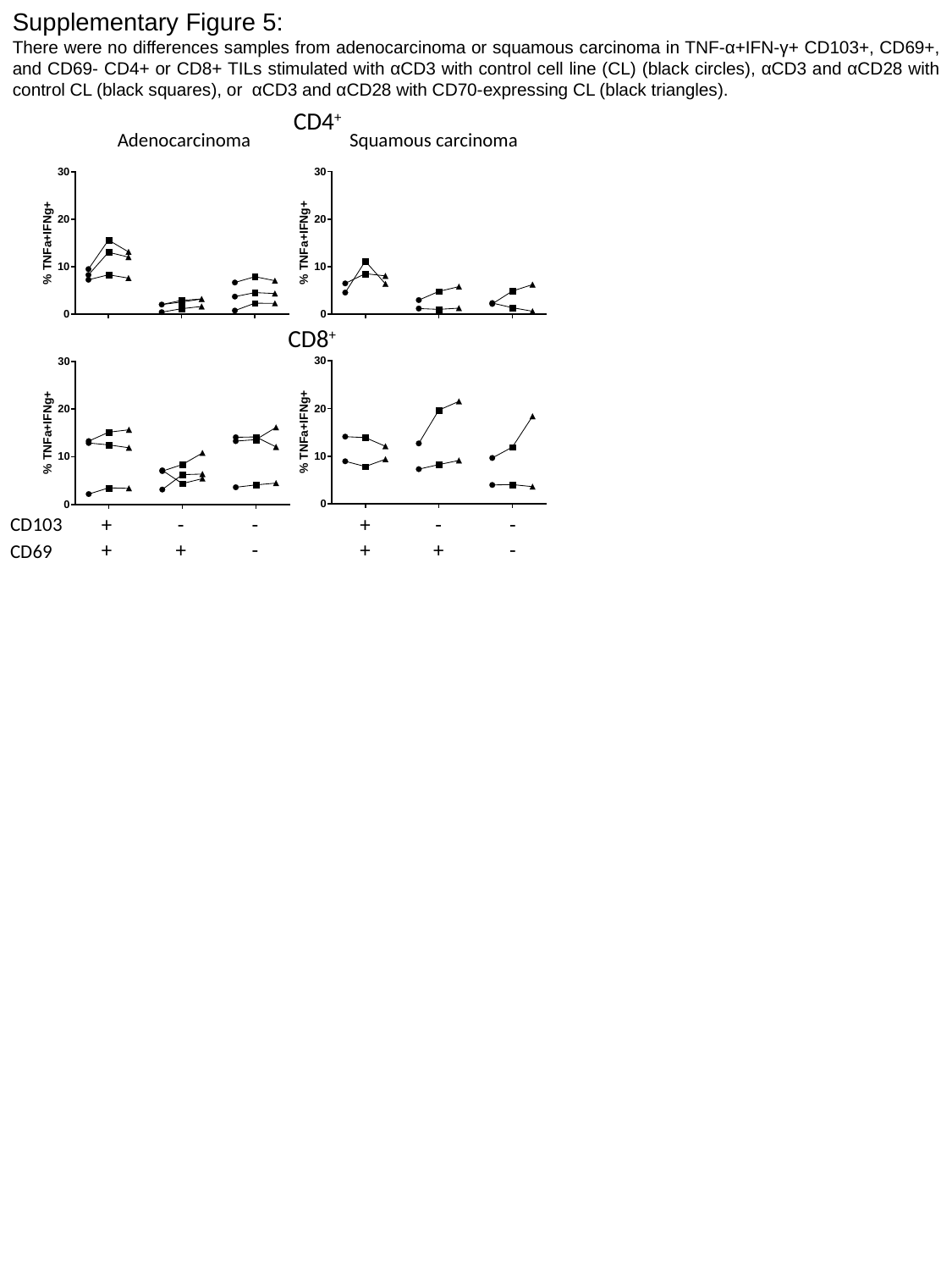

Supplementary Figure 5:
There were no differences samples from adenocarcinoma or squamous carcinoma in TNF-α+IFN-γ+ CD103+, CD69+, and CD69- CD4+ or CD8+ TILs stimulated with αCD3 with control cell line (CL) (black circles), αCD3 and αCD28 with control CL (black squares), or αCD3 and αCD28 with CD70-expressing CL (black triangles).
CD4+
Adenocarcinoma
Squamous carcinoma
CD8+
+
+
-
+
-
-
+
+
-
+
-
-
CD103
CD69
